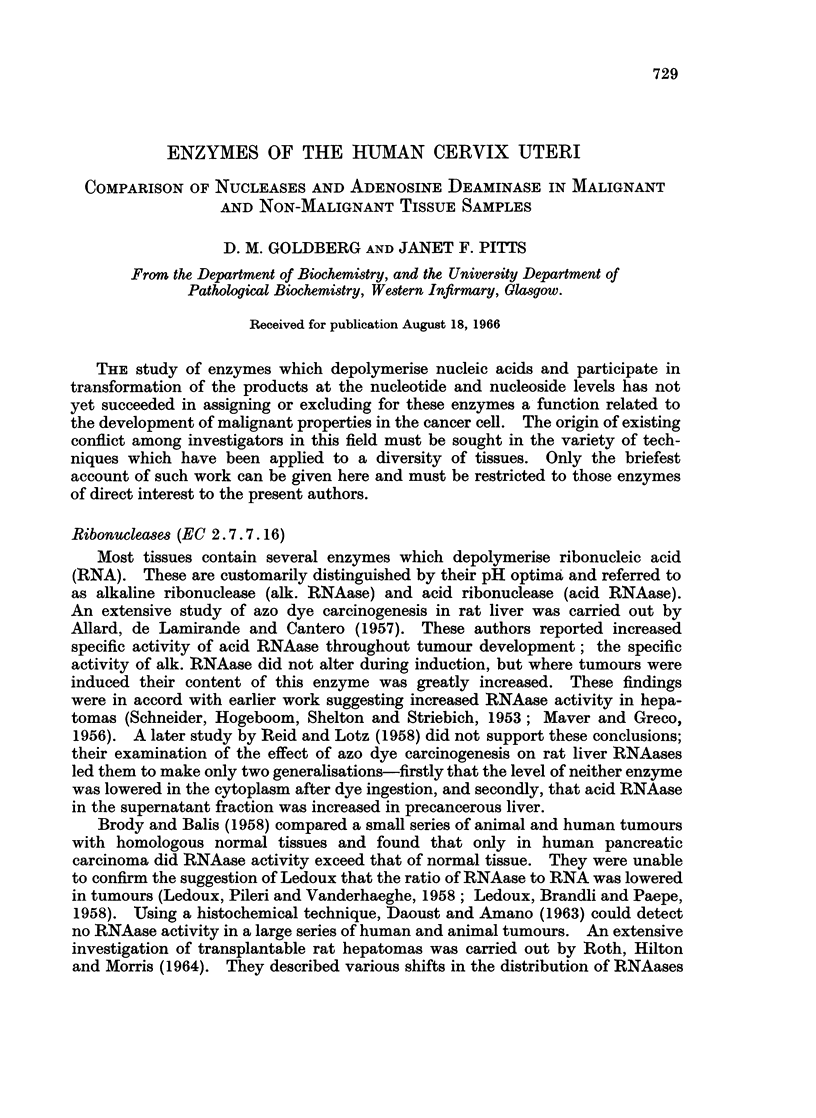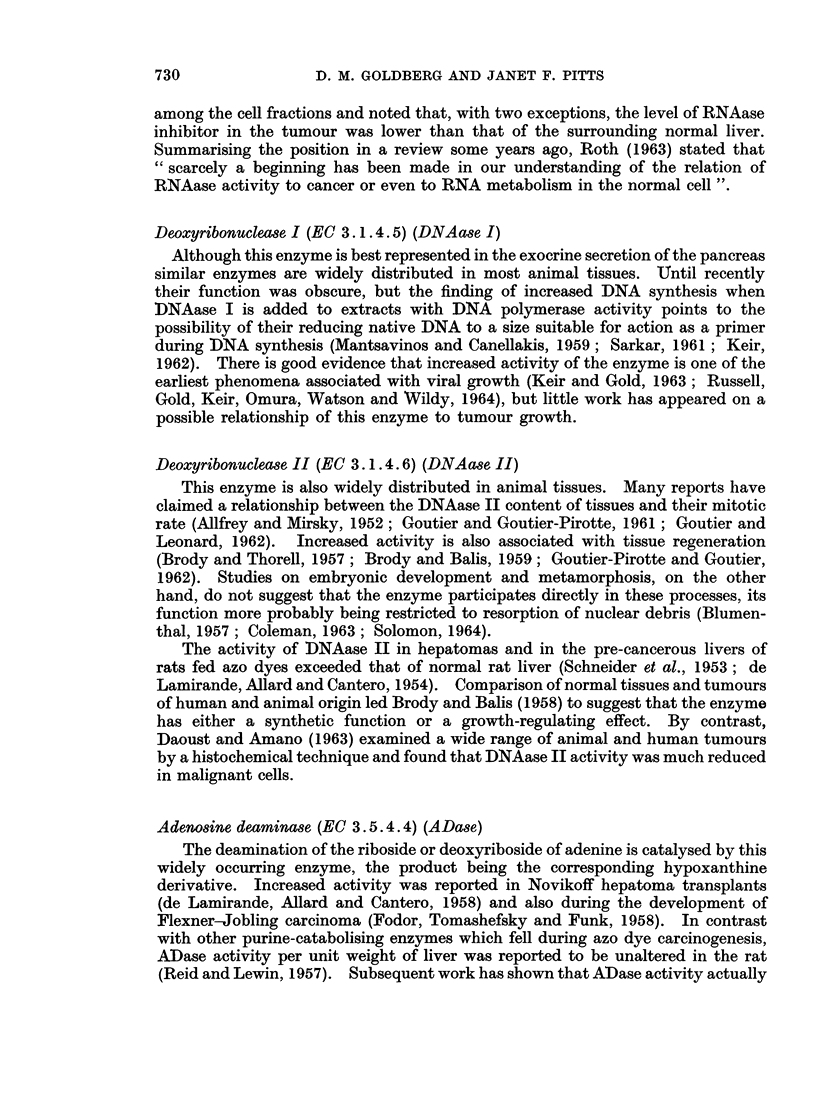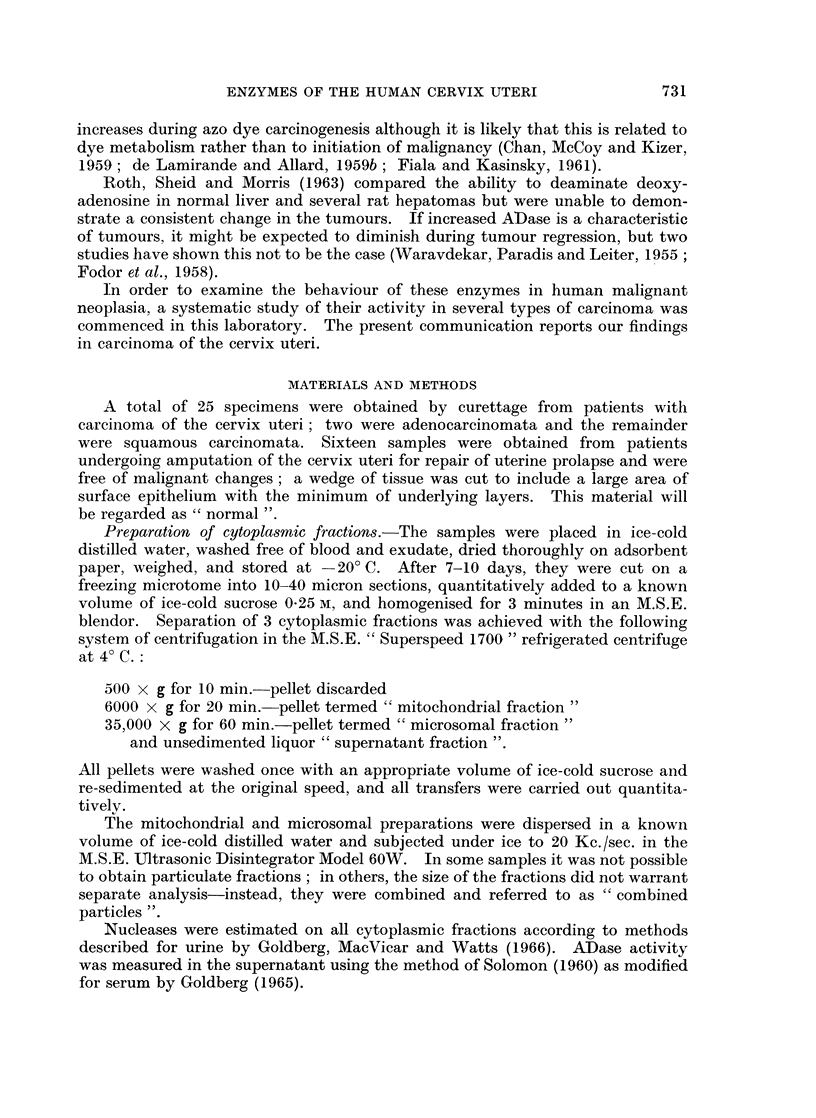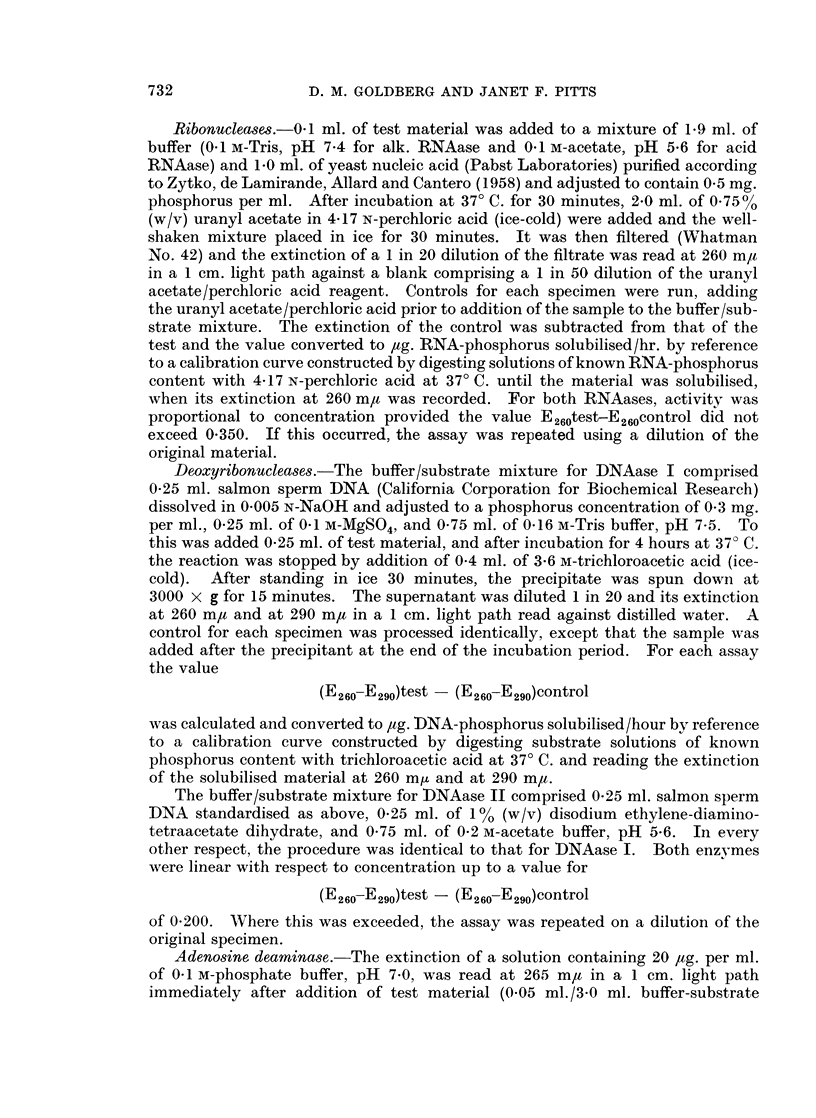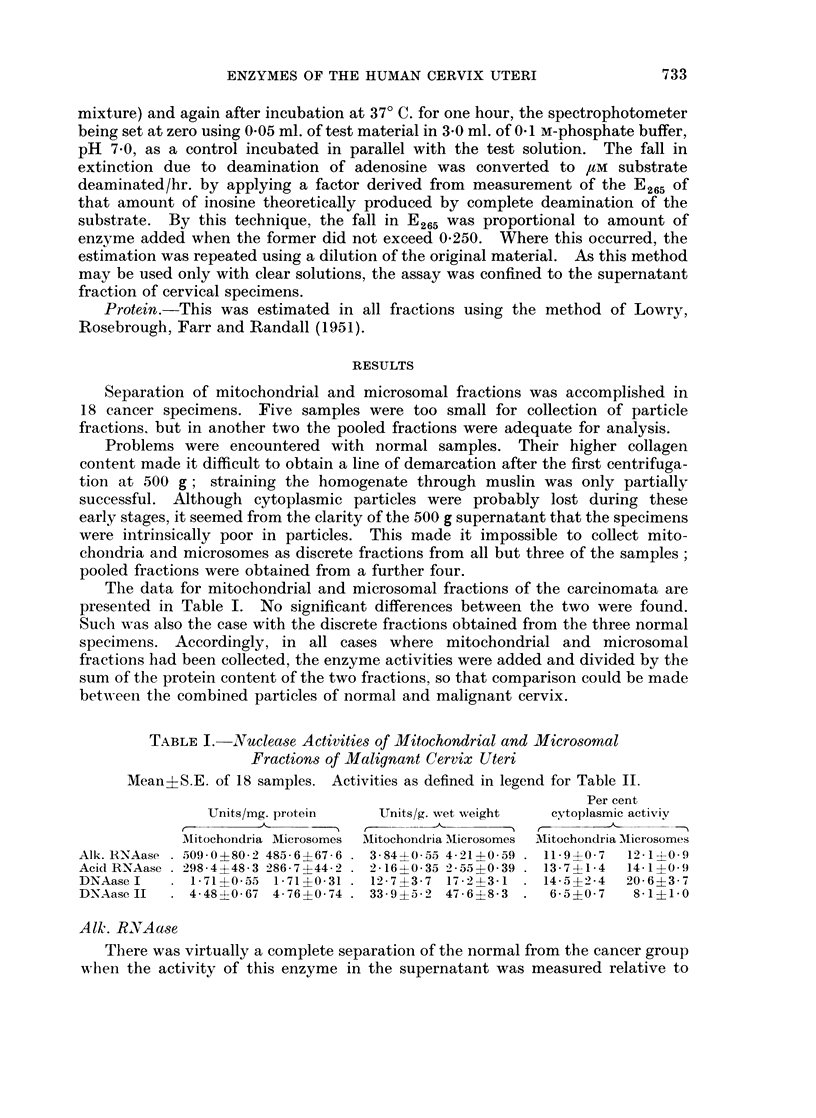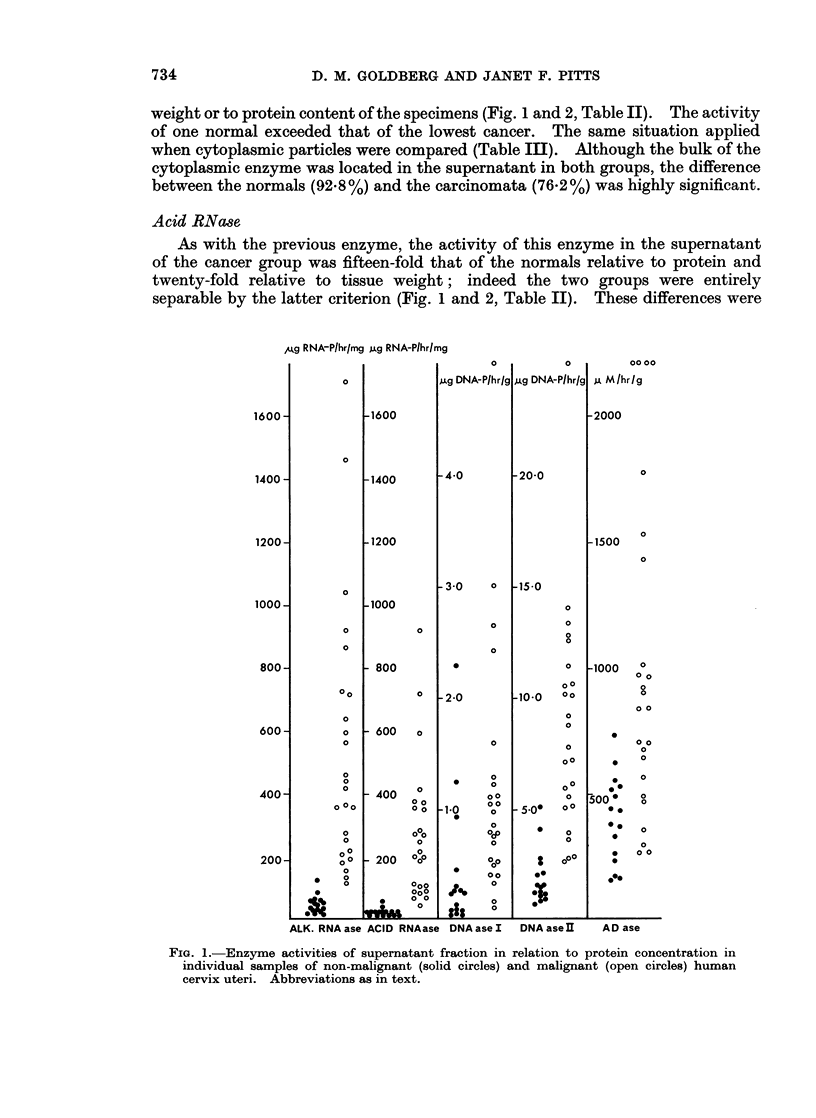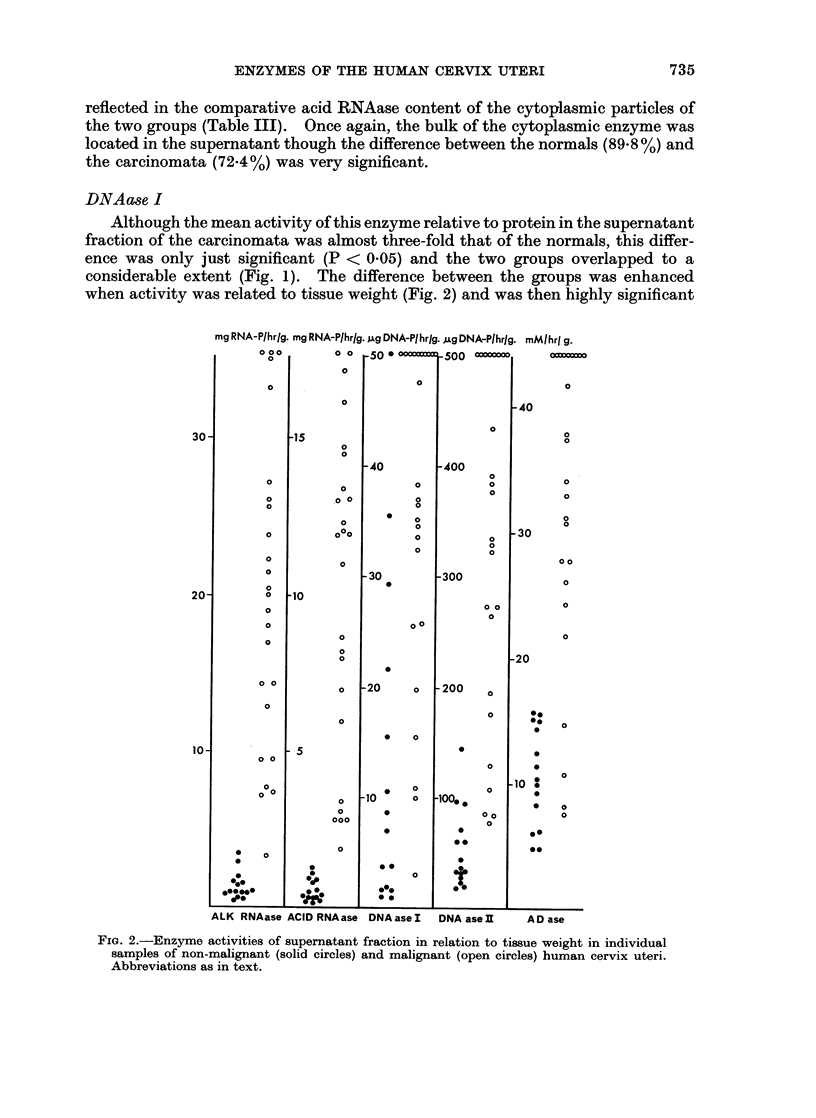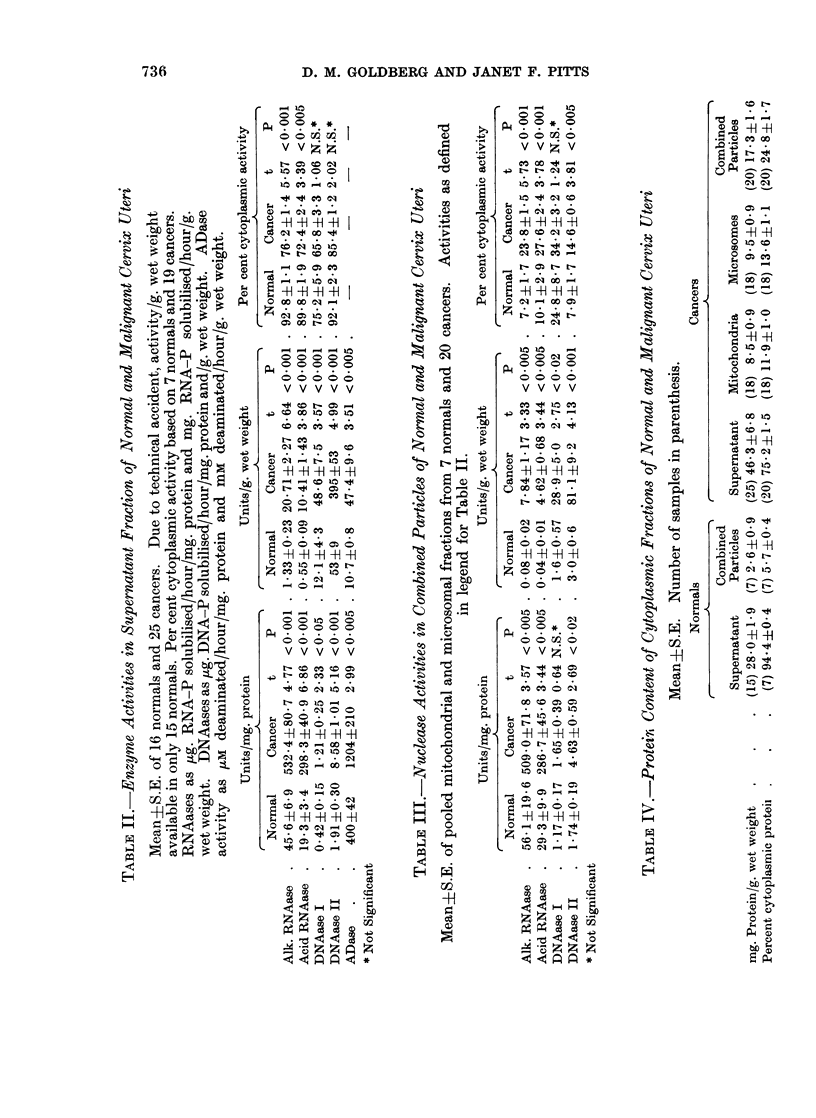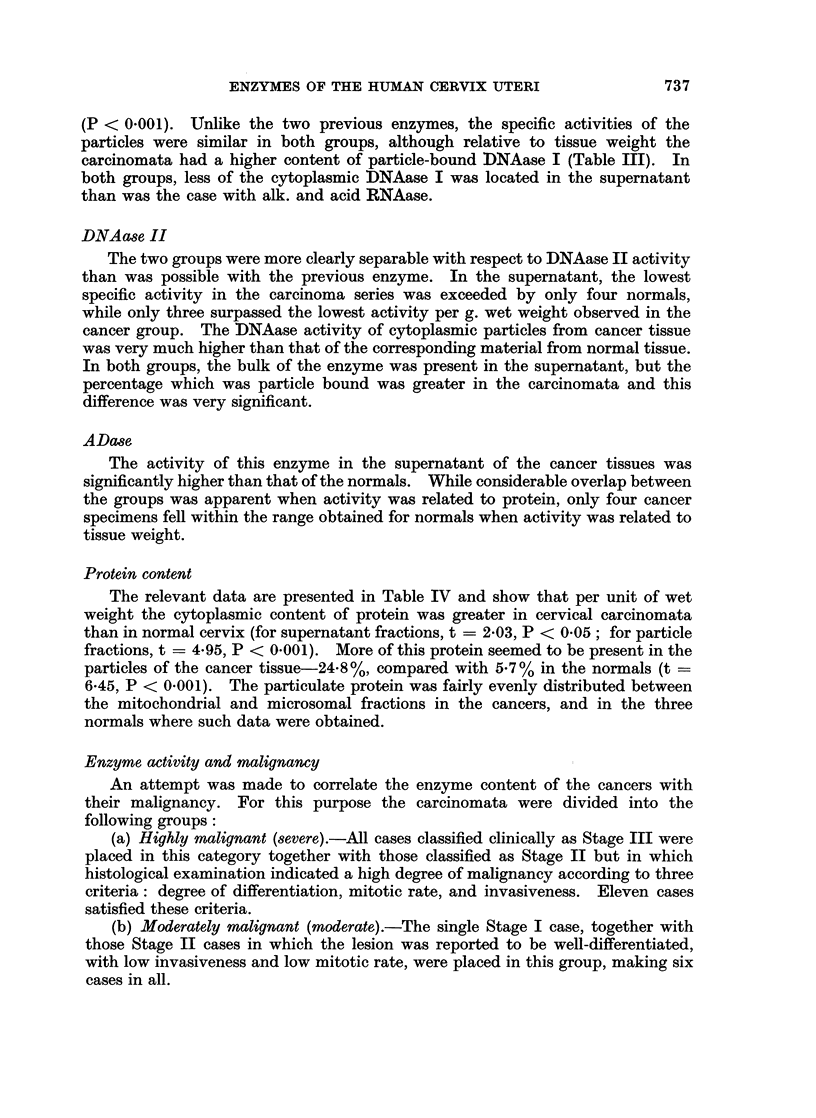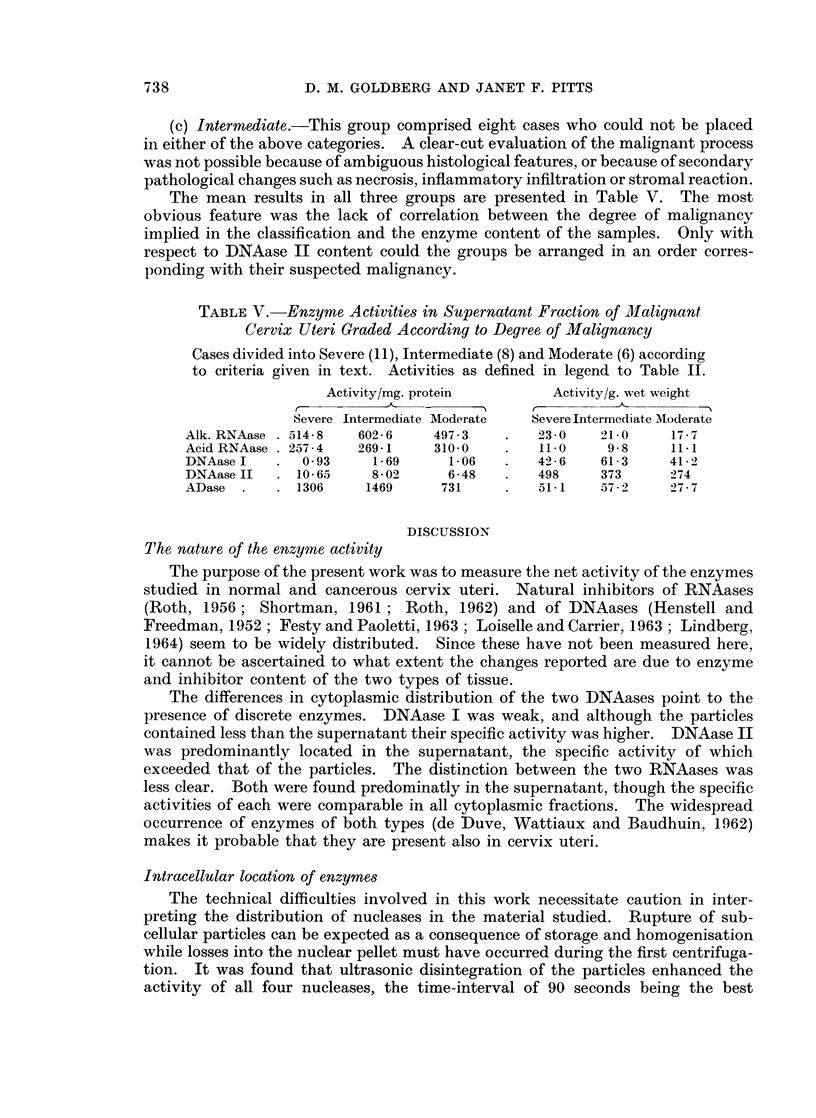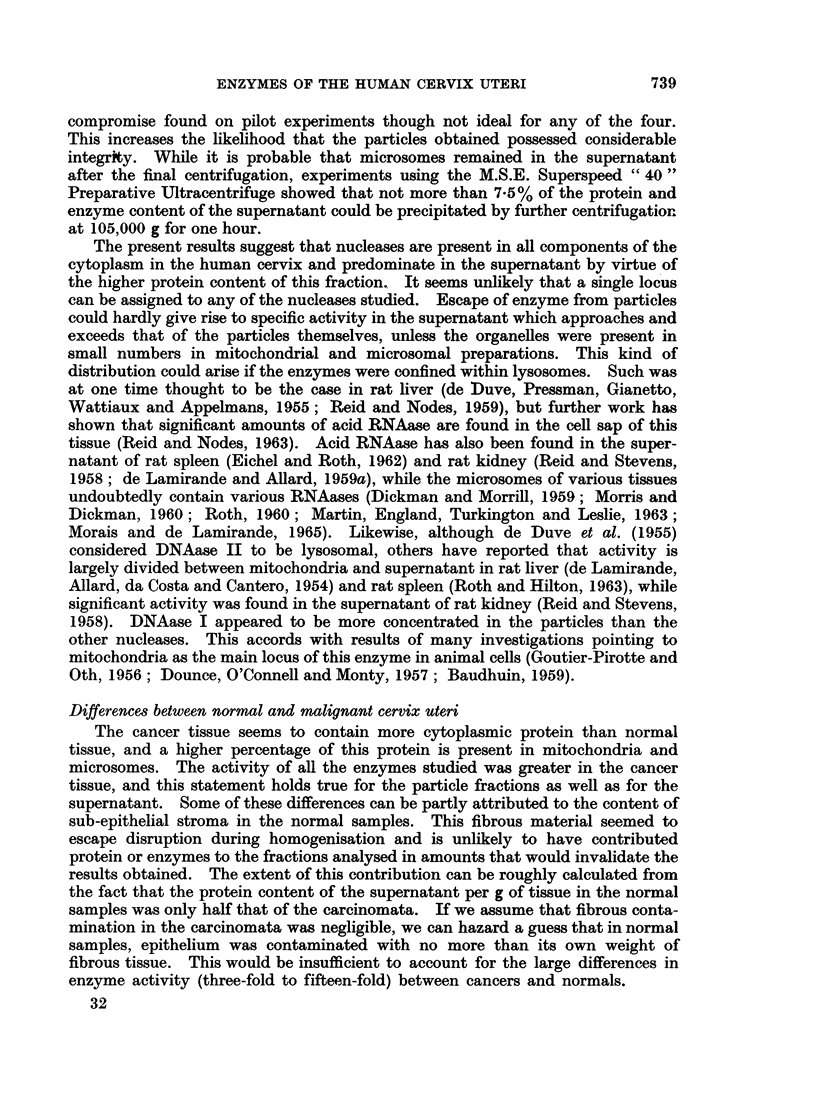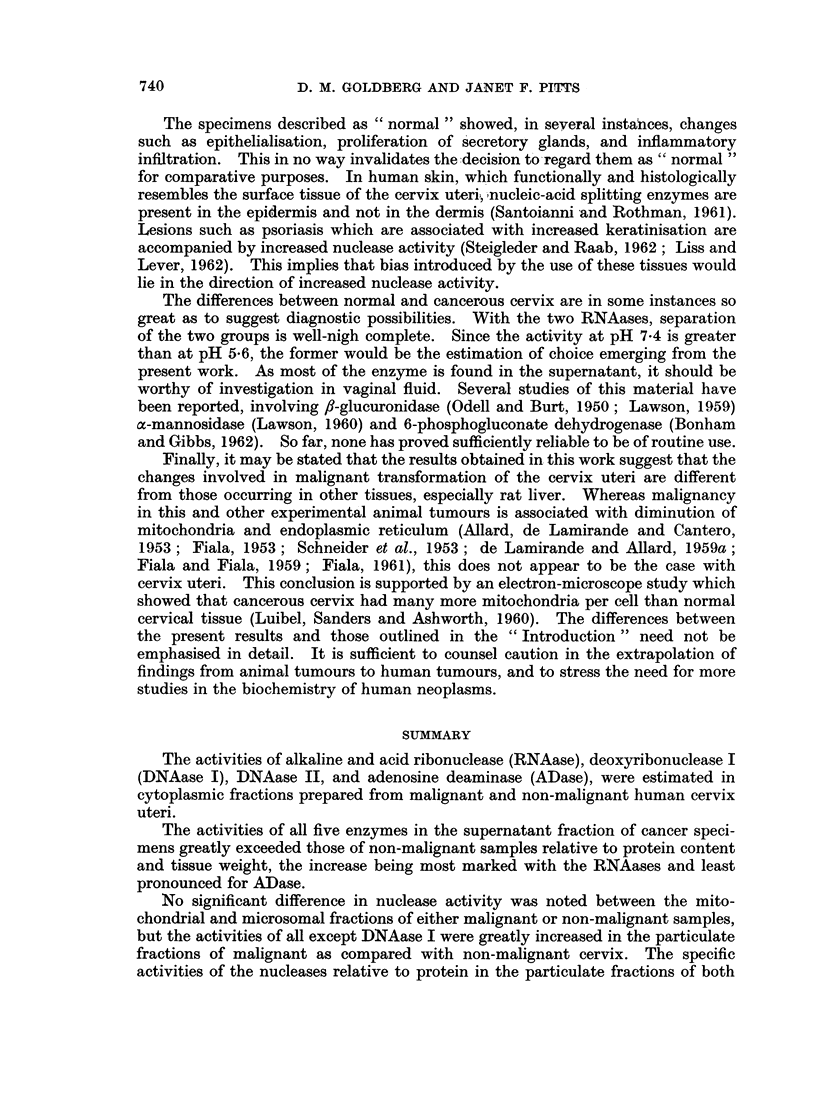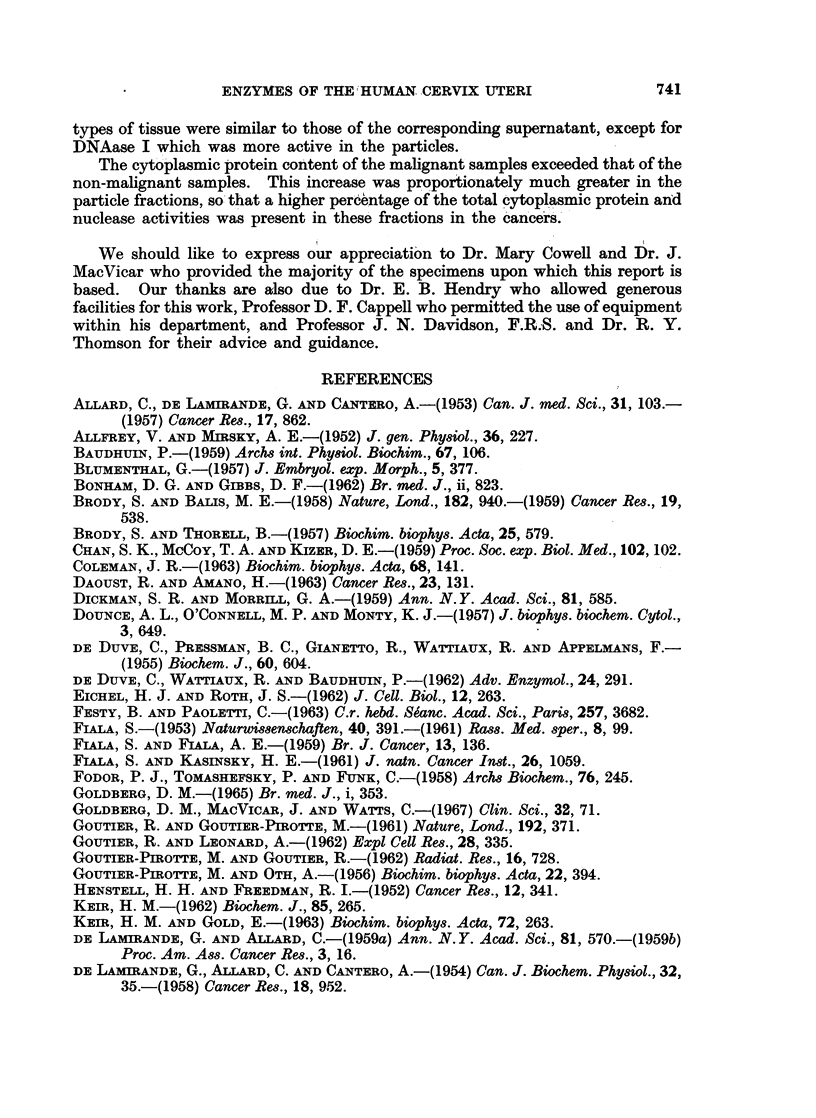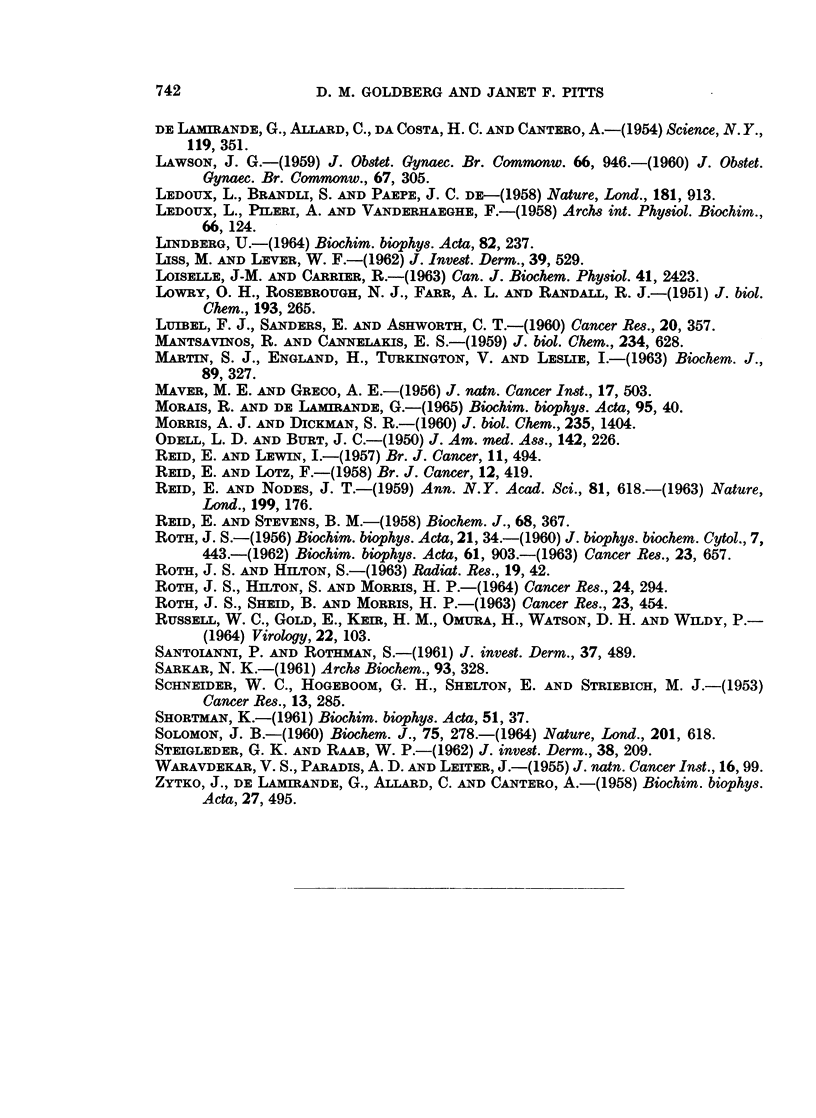# Enzymes of the human cervix uteri. Comparison of nucleases and adenosine deaminase in malignant and non-malignant tissue samples.

**DOI:** 10.1038/bjc.1966.84

**Published:** 1966-12

**Authors:** D. M. Goldberg, J. F. Pitts


### ENZYMES OF THE HUMAN CERVIX UTERI

### Comparison of Nucleases and Adenosine Deaminase in Malignant and Non-Malignant Tissue Samples

D. M. GOLDBERG and JANET F. PITTS

*From the Department of Biochemistry, and the University Department of Pathological Biochemistry, Western Infirmary, Glasgow.*

Received for publication August 18, 1966

THE study of enzymes which depolymerise nucleic acids and participate in transformation of the products at the nucleotide and nucleoside levels has not yet succeeded in assigning or excluding for these enzymes a function related to the development of malignant properties in the cancer cell. The origin of existing conflict among investigators in this field must be sought in the variety of techniques which have been applied to a diversity of tissues. Only the briefest account of such work can be given here and must be restricted to those enzymes of direct interest to the present authors.

*Ribonucleases* (*EC* 2.7.7.16)

Most tissues contain several enzymes which depolymerise ribonucleic acid (RNA). These are customarily distinguished by their pH optima and referred to as alkaline ribonuclease (alk. RNAase) and acid ribonuclease (acid RNAase). An extensive study of azo dye carcinogenesis in rat liver was carried out by Allard, de Lamirande and Cantero (1957). These authors reported increased specific activity of acid RNAase throughout tumour development; the specific activity of alk. RNAase did not alter during induction, but where tumours were induced their content of this enzyme was greatly increased. These findings were in accord with earlier work suggesting increased RNAase activity in hepatomas (Schneider, Hogeboom, Shelton and Striebich, 1953; Maver and Greco, 1956). A later study by Reid and Lotz (1958) did not support these conclusions; their examination of the effect of azo dye carcinogenesis on rat liver RNAases led them to make only two generalisations—firstly that the level of neither enzyme was lowered in the cytoplasm after dye ingestion, and secondly, that acid RNAase in the supernatant fraction was increased in precancerous liver.

Brody and Balis (1958) compared a small series of animal and human tumours with homologous normal tissues and found that only in human pancreatic carcinoma did RNAase activity exceed that of normal tissue. They were unable to confirm the suggestion of Ledoux that the ratio of RNAase to RNA was lowered in tumours (Ledoux, Pileri and Vanderhaeghe, 1958; Ledoux, Brandli and Paepe, 1958). Using a histochemical technique, Daoust and Amano (1963) could detect no RNAase activity in a large series of human and animal tumours. An extensive investigation of transplantable rat hepatomas was carried out by Roth, Hilton and Morris (1964). They described various shifts in the distribution of RNAases among the cell fractions and noted that, with two exceptions, the level of RNAase inhibitor in the tumour was lower than that of the surrounding normal liver. Summarising the position in a review some years ago, Roth (1963) stated that " scarcely a beginning has been made in our understanding of the relation of RNAase activity to cancer or even to RNA metabolism in the normal cell ".

*Deoxyribonuclease I* (*EC* 3.1.4.5) (*DNAase I*)

Although this enzyme is best represented in the exocrine secretion of the pancreas similar enzymes are widely distributed in most animal tissues. Until recently their function was obscure, but the finding of increased DNA synthesis when DNAase I is added to extracts with DNA polymerase activity points to the possibility of their reducing native DNA to a size suitable for action as a primer during DNA synthesis (Mantsavinos and Canellakis, 1959 ; Sarkar, 1961 ; Keir, 1962). There is good evidence that increased activity of the enzyme is one of the earliest phenomena associated with viral growth (Keir and Gold, 1963 ; Russell, Gold, Keir, Omura, Watson and Wildy, 1964), but little work has appeared on a possible relationship of this enzyme to tumour growth.

*Deoxyribonuclease II* (*EC* 3.1.4.6) (*DNAase II*)

This enzyme is also widely distributed in animal tissues. Many reports have claimed a relationship between the DNAase II content of tissues and their mitotic rate (Allfrey and Mirsky, 1952 ; Goutier and Goutier-Pirotte, 1961 ; Goutier and Leonard, 1962). Increased activity is also associated with tissue regeneration (Brody and Thorell, 1957 ; Brody and Balis, 1959 ; Goutier-Pirotte and Goutier, 1962). Studies on embryonic development and metamorphosis, on the other hand, do not suggest that the enzyme participates directly in these processes, its function more probably being restricted to resorption of nuclear debris (Blumenthal, 1957 ; Coleman, 1963 ; Solomon, 1964).

The activity of DNAase II in hepatomas and in the pre-cancerous livers of rats fed azo dyes exceeded that of normal rat liver (Schneider *et al.*, 1953 ; de Lamirande, Allard and Cantero, 1954). Comparison of normal tissues and tumours of human and animal origin led Brody and Balis (1958) to suggest that the enzyme has either a synthetic function or a growth-regulating effect. By contrast, Daoust and Amano (1963) examined a wide range of animal and human tumours by a histochemical technique and found that DNAase II activity was much reduced in malignant cells.

*Adenosine deaminase* (*EC* 3.5.4.4) (*ADase*)

The deamination of the riboside or deoxyriboside of adenine is catalysed by this widely occurring enzyme, the product being the corresponding hypoxanthine derivative. Increased activity was reported in Novikoff hepatoma transplants (de Lamirande, Allard and Cantero, 1958) and also during the development of Flexner–Jobling carcinoma (Fodor, Tomashefsky and Funk, 1958). In contrast with other purine-catabolising enzymes which fell during azo dye carcinogenesis, ADase activity per unit weight of liver was reported to be unaltered in the rat (Reid and Lewin, 1957). Subsequent work has shown that ADase activity actually increases during azo dye carcinogenesis although it is likely that this is related to dye metabolism rather than to initiation of malignancy (Chan, McCoy and Kizer, 1959 ; de Lamirande and Allard, 1959*b* ; Fiala and Kasinsky, 1961).

Roth, Sheid and Morris (1963) compared the ability to deaminate deoxyadenosine in normal liver and several rat hepatomas but were unable to demonstrate a consistent change in the tumours. If increased ADase is a characteristic of tumours, it might be expected to diminish during tumour regression, but two studies have shown this not to be the case (Waravdekar, Paradis and Leiter, 1955 ; Fodor *et al.*, 1958).

In order to examine the behaviour of these enzymes in human malignant neoplasia, a systematic study of their activity in several types of carcinoma was commenced in this laboratory. The present communication reports our findings in carcinoma of the cervix uteri.

### MATERIALS AND METHODS

A total of 25 specimens were obtained by curettage from patients with carcinoma of the cervix uteri ; two were adenocarcinomata and the remainder were squamous carcinomata. Sixteen samples were obtained from patients undergoing amputation of the cervix uteri for repair of uterine prolapse and were free of malignant changes ; a wedge of tissue was cut to include a large area of surface epithelium with the minimum of underlying layers. This material will be regarded as " normal ".

*Preparation of cytoplasmic fractions.*—The samples were placed in ice-cold distilled water, washed free of blood and exudate, dried thoroughly on adsorbent paper, weighed, and stored at $-20°$ C. After 7–10 days, they were cut on a freezing microtome into 10–40 micron sections, quantitatively added to a known volume of ice-cold sucrose 0·25 M, and homogenised for 3 minutes in an M.S.E. blendor. Separation of 3 cytoplasmic fractions was achieved with the following system of centrifugation in the M.S.E. " Superspeed 1700 " refrigerated centrifuge at 4° C. :

500 × **g** for 10 min.—pellet discarded
6000 × **g** for 20 min.—pellet termed " mitochondrial fraction "
35,000 × **g** for 60 min.—pellet termed " microsomal fraction "
and unsedimented liquor " supernatant fraction ".

All pellets were washed once with an appropriate volume of ice-cold sucrose and re-sedimented at the original speed, and all transfers were carried out quantitatively.

The mitochondrial and microsomal preparations were dispersed in a known volume of ice-cold distilled water and subjected under ice to 20 Kc./sec. in the M.S.E. Ultrasonic Disintegrator Model 60W. In some samples it was not possible to obtain particulate fractions ; in others, the size of the fractions did not warrant separate analysis—instead, they were combined and referred to as " combined particles ".

Nucleases were estimated on all cytoplasmic fractions according to methods described for urine by Goldberg, MacVicar and Watts (1966). ADase activity was measured in the supernatant using the method of Solomon (1960) as modified for serum by Goldberg (1965).

*Ribonucleases.*—0·1 ml. of test material was added to a mixture of 1·9 ml. of buffer (0·1 M-Tris, pH 7·4 for alk. RNAase and 0·1 M-acetate, pH 5·6 for acid RNAase) and 1·0 ml. of yeast nucleic acid (Pabst Laboratories) purified according to Zytko, de Lamirande, Allard and Cantero (1958) and adjusted to contain 0·5 mg. phosphorus per ml. After incubation at 37° C. for 30 minutes, 2·0 ml. of 0·75% (w/v) uranyl acetate in 4·17 N-perchloric acid (ice-cold) were added and the well-shaken mixture placed in ice for 30 minutes. It was then filtered (Whatman No. 42) and the extinction of a 1 in 20 dilution of the filtrate was read at 260 mμ in a 1 cm. light path against a blank comprising a 1 in 50 dilution of the uranyl acetate/perchloric acid reagent. Controls for each specimen were run, adding the uranyl acetate/perchloric acid prior to addition of the sample to the buffer/substrate mixture. The extinction of the control was subtracted from that of the test and the value converted to μg. RNA-phosphorus solubilised/hr. by reference to a calibration curve constructed by digesting solutions of known RNA-phosphorus content with 4·17 N-perchloric acid at 37° C. until the material was solubilised, when its extinction at 260 mμ was recorded. For both RNAases, activity was proportional to concentration provided the value $E_{260}$test–$E_{260}$control did not exceed 0·350. If this occurred, the assay was repeated using a dilution of the original material.

*Deoxyribonucleases.*—The buffer/substrate mixture for DNAase I comprised 0·25 ml. salmon sperm DNA (California Corporation for Biochemical Research) dissolved in 0·005 N-NaOH and adjusted to a phosphorus concentration of 0·3 mg. per ml., 0·25 ml. of 0·1 M-$MgSO_4$, and 0·75 ml. of 0·16 M-Tris buffer, pH 7·5. To this was added 0·25 ml. of test material, and after incubation for 4 hours at 37° C. the reaction was stopped by addition of 0·4 ml. of 3·6 M-trichloroacetic acid (ice-cold). After standing in ice 30 minutes, the precipitate was spun down at 3000 × **g** for 15 minutes. The supernatant was diluted 1 in 20 and its extinction at 260 mμ and at 290 mμ in a 1 cm. light path read against distilled water. A control for each specimen was processed identically, except that the sample was added after the precipitant at the end of the incubation period. For each assay the value

$$(E_{260}–E_{290})\text{test} - (E_{260}–E_{290})\text{control}$$

was calculated and converted to μg. DNA-phosphorus solubilised/hour by reference to a calibration curve constructed by digesting substrate solutions of known phosphorus content with trichloroacetic acid at 37° C. and reading the extinction of the solubilised material at 260 mμ and at 290 mμ.

The buffer/substrate mixture for DNAase II comprised 0·25 ml. salmon sperm DNA standardised as above, 0·25 ml. of 1% (w/v) disodium ethylene-diamino-tetraacetate dihydrate, and 0·75 ml. of 0·2 M-acetate buffer, pH 5·6. In every other respect, the procedure was identical to that for DNAase I. Both enzymes were linear with respect to concentration up to a value for

$$(E_{260}–E_{290})\text{test} - (E_{260}–E_{290})\text{control}$$

of 0·200. Where this was exceeded, the assay was repeated on a dilution of the original specimen.

*Adenosine deaminase.*—The extinction of a solution containing 20 μg. per ml. of 0·1 M-phosphate buffer, pH 7·0, was read at 265 mμ in a 1 cm. light path immediately after addition of test material (0·05 ml./3·0 ml. buffer-substrate mixture) and again after incubation at 37° C. for one hour, the spectrophotometer being set at zero using 0·05 ml. of test material in 3·0 ml. of 0·1 M-phosphate buffer, pH 7·0, as a control incubated in parallel with the test solution. The fall in extinction due to deamination of adenosine was converted to $\mu$M substrate deaminated/hr. by applying a factor derived from measurement of the $E_{265}$ of that amount of inosine theoretically produced by complete deamination of the substrate. By this technique, the fall in $E_{265}$ was proportional to amount of enzyme added when the former did not exceed 0·250. Where this occurred, the estimation was repeated using a dilution of the original material. As this method may be used only with clear solutions, the assay was confined to the supernatant fraction of cervical specimens.

*Protein.*—This was estimated in all fractions using the method of Lowry, Rosebrough, Farr and Randall (1951).

### RESULTS

Separation of mitochondrial and microsomal fractions was accomplished in 18 cancer specimens. Five samples were too small for collection of particle fractions, but in another two the pooled fractions were adequate for analysis.

Problems were encountered with normal samples. Their higher collagen content made it difficult to obtain a line of demarcation after the first centrifugation at 500 **g** ; straining the homogenate through muslin was only partially successful. Although cytoplasmic particles were probably lost during these early stages, it seemed from the clarity of the 500 **g** supernatant that the specimens were intrinsically poor in particles. This made it impossible to collect mitochondria and microsomes as discrete fractions from all but three of the samples ; pooled fractions were obtained from a further four.

The data for mitochondrial and microsomal fractions of the carcinomata are presented in Table I. No significant differences between the two were found. Such was also the case with the discrete fractions obtained from the three normal specimens. Accordingly, in all cases where mitochondrial and microsomal fractions had been collected, the enzyme activities were added and divided by the sum of the protein content of the two fractions, so that comparison could be made between the combined particles of normal and malignant cervix.

TABLE I.—*Nuclease Activities of Mitochondrial and Microsomal Fractions of Malignant Cervix Uteri*

Mean±S.E. of 18 samples. Activities as defined in legend for Table II.

| | Units/mg. protein | | Units/g. wet weight | | Per cent cytoplasmic activiy | |
|---|---|---|---|---|---|---|
| | Mitochondria | Microsomes | Mitochondria | Microsomes | Mitochondria | Microsomes |
| Alk. RNAase | 509·0±80·2 | 485·6±67·6 | 3·84±0·55 | 4·21±0·59 | 11·9±0·7 | 12·1±0·9 |
| Acid RNAase | 298·4±48·3 | 286·7±44·2 | 2·16±0·35 | 2·55±0·39 | 13·7±1·4 | 14·1±0·9 |
| DNAase I | 1·71±0·55 | 1·71±0·31 | 12·7±3·7 | 17·2±3·1 | 14·5±2·4 | 20·6±3·7 |
| DNAase II | 4·48±0·67 | 4·76±0·74 | 33·9±5·2 | 47·6±8·3 | 6·5±0·7 | 8·1±1·0 |

### *Alk. RNAase*

There was virtually a complete separation of the normal from the cancer group when the activity of this enzyme in the supernatant was measured relative to weight or to protein content of the specimens (Fig. 1 and 2, Table II). The activity of one normal exceeded that of the lowest cancer. The same situation applied when cytoplasmic particles were compared (Table III). Although the bulk of the cytoplasmic enzyme was located in the supernatant in both groups, the difference between the normals (92·8 %) and the carcinomata (76·2 %) was highly significant.

*Acid RNase*

As with the previous enzyme, the activity of this enzyme in the supernatant of the cancer group was fifteen-fold that of the normals relative to protein and twenty-fold relative to tissue weight; indeed the two groups were entirely separable by the latter criterion (Fig. 1 and 2, Table II). These differences were

FIG. 1.—Enzyme activities of supernatant fraction in relation to protein concentration in individual samples of non-malignant (solid circles) and malignant (open circles) human cervix uteri. Abbreviations as in text.

reflected in the comparative acid RNAase content of the cytoplasmic particles of the two groups (Table III). Once again, the bulk of the cytoplasmic enzyme was located in the supernatant though the difference between the normals (89·8 %) and the carcinomata (72·4 %) was very significant.

*DNAase I*

Although the mean activity of this enzyme relative to protein in the supernatant fraction of the carcinomata was almost three-fold that of the normals, this difference was only just significant ($P < 0{\cdot}05$) and the two groups overlapped to a considerable extent (Fig. 1). The difference between the groups was enhanced when activity was related to tissue weight (Fig. 2) and was then highly significant

FIG. 2.—Enzyme activities of supernatant fraction in relation to tissue weight in individual samples of non-malignant (solid circles) and malignant (open circles) human cervix uteri. Abbreviations as in text.

TABLE II.—*Enzyme Activities in Supernatant Fraction of Normal and Malignant Cervix Uteri*

Mean±S.E. of 16 normals and 25 cancers. Due to technical accident, activity/g. wet weight available in only 15 normals. Per cent cytoplasmic activity based on 7 normals and 19 cancers. RNAases as μg. RNA–P solubilised/hour/mg. protein and mg. RNA–P solubilised/hour/g. wet weight. DNAases as μg. DNA–P solubilised/hour/mg. protein and/g. wet weight. ADase activity as μM deaminated/hour/mg. protein and mM deaminated/hour/g. wet weight.

| | Units/mg. protein | | | | Units/g. wet weight | | | | Per cent cytoplasmic activity | | | |
|---|---|---|---|---|---|---|---|---|---|---|---|---|
| | Normal | Cancer | t | P | Normal | Cancer | t | P | Normal | Cancer | t | P |
| Alk. RNAase | 45·6±6·9 | 532·4±80·7 | 4·77 | <0·001 | 1·33±0·23 | 20·71±2·27 | 6·64 | <0·001 | 92·8±1·1 | 76·2±1·4 | 5·57 | <0·001 |
| Acid RNAase | 19·3±3·4 | 298·3±40·9 | 6·86 | <0·001 | 0·55±0·09 | 10·41±1·43 | 3·86 | <0·001 | 89·8±1·9 | 72·4±2·4 | 3·39 | <0·005 |
| DNAase I | 0·42±0·15 | 1·21±0·25 | 2·33 | <0·05 | 12·1±4·3 | 48·6±7·5 | 3·57 | <0·001 | 75·2±5·9 | 65·8±3·3 | 1·06 | N.S.* |
| DNAase II | 1·91±0·30 | 8·58±1·01 | 5·16 | <0·001 | 53±9 | 395±53 | 4·99 | <0·001 | 92·1±2·3 | 85·4±1·2 | 2·02 | N.S.* |
| ADase | 400±42 | 1204±210 | 2·99 | <0·005 | 10·7±0·8 | 47·4±9·6 | 3·51 | <0·005 | — | — | — | — |

* Not Significant

TABLE III.—*Nuclease Activities in Combined Particles of Normal and Malignant Cervix Uteri*

Mean±S.E. of pooled mitochondrial and microsomal fractions from 7 normals and 20 cancers. Activities as defined in legend for Table II.

| | Units/mg. protein | | | | Units/g. wet weight | | | | Per cent cytoplasmic activity | | | |
|---|---|---|---|---|---|---|---|---|---|---|---|---|
| | Normal | Cancer | t | P | Normal | Cancer | t | P | Normal | Cancer | t | P |
| Alk. RNAase | 56·1±19·6 | 509·0±71·8 | 3·57 | <0·005 | 0·08±0·02 | 7·84±1·17 | 3·33 | <0·005 | 7·2±1·7 | 23·8±1·5 | 5·73 | <0·001 |
| Acid RNAase | 29·3±9·9 | 286·7±45·6 | 3·44 | <0·005 | 0·04±0·01 | 4·62±0·68 | 3·44 | <0·005 | 10·1±2·9 | 27·6±2·4 | 3·78 | <0·001 |
| DNAase I | 1·17±0·17 | 1·65±0·39 | 0·64 | N.S.* | 1·6±0·57 | 28·9±5·0 | 2·75 | <0·02 | 24·8±8·7 | 34·2±3·2 | 1·24 | N.S.* |
| DNAase II | 1·74±0·19 | 4·63±0·59 | 2·69 | <0·02 | 3·0±0·6 | 81·1±9·2 | 4·13 | <0·001 | 7·9±1·7 | 14·6±0·6 | 3·81 | <0·005 |

* Not Significant

TABLE IV.—*Protein Content of Cytoplasmic Fractions of Normal and Malignant Cervix Uteri*

Mean±S.E. Number of samples in parenthesis.

| | Normals | | Cancers | | | |
|---|---|---|---|---|---|---|
| | Supernatant | Combined Particles | Supernatant | Mitochondria | Microsomes | Combined Particles |
| mg. Protein/g. wet weight | (15) 28·0±1·9 | (7) 2·6±0·9 | (25) 46·3±6·8 | (18) 8·5±0·9 | (18) 9·5±0·9 | (20) 17·3±1·6 |
| Percent cytoplasmic protei[illegible] | (7) 94·4±0·4 | (7) 5·7±0·4 | (20) 75·2±1·5 | (18) 11·9±1·0 | (18) 13·6±1·1 | (20) 24·8±1·7 |

($P < 0{\cdot}001$). Unlike the two previous enzymes, the specific activities of the particles were similar in both groups, although relative to tissue weight the carcinomata had a higher content of particle-bound DNAase I (Table III). In both groups, less of the cytoplasmic DNAase I was located in the supernatant than was the case with alk. and acid RNAase.

*DNAase II*

The two groups were more clearly separable with respect to DNAase II activity than was possible with the previous enzyme. In the supernatant, the lowest specific activity in the carcinoma series was exceeded by only four normals, while only three surpassed the lowest activity per g. wet weight observed in the cancer group. The DNAase activity of cytoplasmic particles from cancer tissue was very much higher than that of the corresponding material from normal tissue. In both groups, the bulk of the enzyme was present in the supernatant, but the percentage which was particle bound was greater in the carcinomata and this difference was very significant.

*ADase*

The activity of this enzyme in the supernatant of the cancer tissues was significantly higher than that of the normals. While considerable overlap between the groups was apparent when activity was related to protein, only four cancer specimens fell within the range obtained for normals when activity was related to tissue weight.

*Protein content*

The relevant data are presented in Table IV and show that per unit of wet weight the cytoplasmic content of protein was greater in cervical carcinomata than in normal cervix (for supernatant fractions, $t = 2{\cdot}03$, $P < 0{\cdot}05$ ; for particle fractions, $t = 4{\cdot}95$, $P < 0{\cdot}001$). More of this protein seemed to be present in the particles of the cancer tissue—24·8%, compared with 5·7% in the normals ($t = 6{\cdot}45$, $P < 0{\cdot}001$). The particulate protein was fairly evenly distributed between the mitochondrial and microsomal fractions in the cancers, and in the three normals where such data were obtained.

*Enzyme activity and malignancy*

An attempt was made to correlate the enzyme content of the cancers with their malignancy. For this purpose the carcinomata were divided into the following groups :

(a) *Highly malignant* (*severe*).—All cases classified clinically as Stage III were placed in this category together with those classified as Stage II but in which histological examination indicated a high degree of malignancy according to three criteria : degree of differentiation, mitotic rate, and invasiveness. Eleven cases satisfied these criteria.

(b) *Moderately malignant* (*moderate*).—The single Stage I case, together with those Stage II cases in which the lesion was reported to be well-differentiated, with low invasiveness and low mitotic rate, were placed in this group, making six cases in all.

(c) *Intermediate.*—This group comprised eight cases who could not be placed in either of the above categories. A clear-cut evaluation of the malignant process was not possible because of ambiguous histological features, or because of secondary pathological changes such as necrosis, inflammatory infiltration or stromal reaction.

The mean results in all three groups are presented in Table V. The most obvious feature was the lack of correlation between the degree of malignancy implied in the classification and the enzyme content of the samples. Only with respect to DNAase II content could the groups be arranged in an order corresponding with their suspected malignancy.

TABLE V.—*Enzyme Activities in Supernatant Fraction of Malignant Cervix Uteri Graded According to Degree of Malignancy*

Cases divided into Severe (11), Intermediate (8) and Moderate (6) according to criteria given in text. Activities as defined in legend to Table II.

| | Activity/mg. protein | | | Activity/g. wet weight | | |
|---|---|---|---|---|---|---|
| | Severe | Intermediate | Moderate | Severe | Intermediate | Moderate |
| Alk. RNAase | 514·8 | 602·6 | 497·3 | 23·0 | 21·0 | 17·7 |
| Acid RNAase | 257·4 | 269·1 | 310·0 | 11·0 | 9·8 | 11·1 |
| DNAase I | 0·93 | 1·69 | 1·06 | 42·6 | 61·3 | 41·2 |
| DNAase II | 10·65 | 8·02 | 6·48 | 498 | 373 | 274 |
| ADase | 1306 | 1469 | 731 | 51·1 | 57·2 | 27·7 |

### DISCUSSION

### *The nature of the enzyme activity*

The purpose of the present work was to measure the net activity of the enzymes studied in normal and cancerous cervix uteri. Natural inhibitors of RNAases (Roth, 1956; Shortman, 1961; Roth, 1962) and of DNAases (Henstell and Freedman, 1952; Festy and Paoletti, 1963; Loiselle and Carrier, 1963; Lindberg, 1964) seem to be widely distributed. Since these have not been measured here, it cannot be ascertained to what extent the changes reported are due to enzyme and inhibitor content of the two types of tissue.

The differences in cytoplasmic distribution of the two DNAases point to the presence of discrete enzymes. DNAase I was weak, and although the particles contained less than the supernatant their specific activity was higher. DNAase II was predominantly located in the supernatant, the specific activity of which exceeded that of the particles. The distinction between the two RNAases was less clear. Both were found predominatly in the supernatant, though the specific activities of each were comparable in all cytoplasmic fractions. The widespread occurrence of enzymes of both types (de Duve, Wattiaux and Baudhuin, 1962) makes it probable that they are present also in cervix uteri.

### *Intracellular location of enzymes*

The technical difficulties involved in this work necessitate caution in interpreting the distribution of nucleases in the material studied. Rupture of subcellular particles can be expected as a consequence of storage and homogenisation while losses into the nuclear pellet must have occurred during the first centrifugation. It was found that ultrasonic disintegration of the particles enhanced the activity of all four nucleases, the time-interval of 90 seconds being the best compromise found on pilot experiments though not ideal for any of the four. This increases the likelihood that the particles obtained possessed considerable integrity. While it is probable that microsomes remained in the supernatant after the final centrifugation, experiments using the M.S.E. Superspeed " 40 " Preparative Ultracentrifuge showed that not more than 7·5% of the protein and enzyme content of the supernatant could be precipitated by further centrifugation at 105,000 g for one hour.

The present results suggest that nucleases are present in all components of the cytoplasm in the human cervix and predominate in the supernatant by virtue of the higher protein content of this fraction. It seems unlikely that a single locus can be assigned to any of the nucleases studied. Escape of enzyme from particles could hardly give rise to specific activity in the supernatant which approaches and exceeds that of the particles themselves, unless the organelles were present in small numbers in mitochondrial and microsomal preparations. This kind of distribution could arise if the enzymes were confined within lysosomes. Such was at one time thought to be the case in rat liver (de Duve, Pressman, Gianetto, Wattiaux and Appelmans, 1955 ; Reid and Nodes, 1959), but further work has shown that significant amounts of acid RNAase are found in the cell sap of this tissue (Reid and Nodes, 1963). Acid RNAase has also been found in the supernatant of rat spleen (Eichel and Roth, 1962) and rat kidney (Reid and Stevens, 1958 ; de Lamirande and Allard, 1959*a*), while the microsomes of various tissues undoubtedly contain various RNAases (Dickman and Morrill, 1959 ; Morris and Dickman, 1960 ; Roth, 1960 ; Martin, England, Turkington and Leslie, 1963 ; Morais and de Lamirande, 1965). Likewise, although de Duve *et al.* (1955) considered DNAase II to be lysosomal, others have reported that activity is largely divided between mitochondria and supernatant in rat liver (de Lamirande, Allard, da Costa and Cantero, 1954) and rat spleen (Roth and Hilton, 1963), while significant activity was found in the supernatant of rat kidney (Reid and Stevens, 1958). DNAase I appeared to be more concentrated in the particles than the other nucleases. This accords with results of many investigations pointing to mitochondria as the main locus of this enzyme in animal cells (Goutier-Pirotte and Oth, 1956 ; Dounce, O'Connell and Monty, 1957 ; Baudhuin, 1959).

*Differences between normal and malignant cervix uteri*

The cancer tissue seems to contain more cytoplasmic protein than normal tissue, and a higher percentage of this protein is present in mitochondria and microsomes. The activity of all the enzymes studied was greater in the cancer tissue, and this statement holds true for the particle fractions as well as for the supernatant. Some of these differences can be partly attributed to the content of sub-epithelial stroma in the normal samples. This fibrous material seemed to escape disruption during homogenisation and is unlikely to have contributed protein or enzymes to the fractions analysed in amounts that would invalidate the results obtained. The extent of this contribution can be roughly calculated from the fact that the protein content of the supernatant per g of tissue in the normal samples was only half that of the carcinomata. If we assume that fibrous contamination in the carcinomata was negligible, we can hazard a guess that in normal samples, epithelium was contaminated with no more than its own weight of fibrous tissue. This would be insufficient to account for the large differences in enzyme activity (three-fold to fifteen-fold) between cancers and normals.

The specimens described as " normal " showed, in several instances, changes such as epithelialisation, proliferation of secretory glands, and inflammatory infiltration. This in no way invalidates the decision to regard them as " normal " for comparative purposes. In human skin, which functionally and histologically resembles the surface tissue of the cervix uteri, nucleic-acid splitting enzymes are present in the epidermis and not in the dermis (Santoianni and Rothman, 1961). Lesions such as psoriasis which are associated with increased keratinisation are accompanied by increased nuclease activity (Steigleder and Raab, 1962 ; Liss and Lever, 1962). This implies that bias introduced by the use of these tissues would lie in the direction of increased nuclease activity.

The differences between normal and cancerous cervix are in some instances so great as to suggest diagnostic possibilities. With the two RNAases, separation of the two groups is well-nigh complete. Since the activity at pH 7·4 is greater than at pH 5·6, the former would be the estimation of choice emerging from the present work. As most of the enzyme is found in the supernatant, it should be worthy of investigation in vaginal fluid. Several studies of this material have been reported, involving $\beta$-glucuronidase (Odell and Burt, 1950 ; Lawson, 1959) $\alpha$-mannosidase (Lawson, 1960) and 6-phosphogluconate dehydrogenase (Bonham and Gibbs, 1962). So far, none has proved sufficiently reliable to be of routine use.

Finally, it may be stated that the results obtained in this work suggest that the changes involved in malignant transformation of the cervix uteri are different from those occurring in other tissues, especially rat liver. Whereas malignancy in this and other experimental animal tumours is associated with diminution of mitochondria and endoplasmic reticulum (Allard, de Lamirande and Cantero, 1953 ; Fiala, 1953 ; Schneider *et al.*, 1953 ; de Lamirande and Allard, 1959*a* ; Fiala and Fiala, 1959 ; Fiala, 1961), this does not appear to be the case with cervix uteri. This conclusion is supported by an electron-microscope study which showed that cancerous cervix had many more mitochondria per cell than normal cervical tissue (Luibel, Sanders and Ashworth, 1960). The differences between the present results and those outlined in the " Introduction " need not be emphasised in detail. It is sufficient to counsel caution in the extrapolation of findings from animal tumours to human tumours, and to stress the need for more studies in the biochemistry of human neoplasms.

### SUMMARY

The activities of alkaline and acid ribonuclease (RNAase), deoxyribonuclease I (DNAase I), DNAase II, and adenosine deaminase (ADase), were estimated in cytoplasmic fractions prepared from malignant and non-malignant human cervix uteri.

The activities of all five enzymes in the supernatant fraction of cancer specimens greatly exceeded those of non-malignant samples relative to protein content and tissue weight, the increase being most marked with the RNAases and least pronounced for ADase.

No significant difference in nuclease activity was noted between the mitochondrial and microsomal fractions of either malignant or non-malignant samples, but the activities of all except DNAase I were greatly increased in the particulate fractions of malignant as compared with non-malignant cervix. The specific activities of the nucleases relative to protein in the particulate fractions of both types of tissue were similar to those of the corresponding supernatant, except for DNAase I which was more active in the particles.

The cytoplasmic protein content of the malignant samples exceeded that of the non-malignant samples. This increase was proportionately much greater in the particle fractions, so that a higher percentage of the total cytoplasmic protein and nuclease activities was present in these fractions in the cancers.

We should like to express our appreciation to Dr. Mary Cowell and Dr. J. MacVicar who provided the majority of the specimens upon which this report is based. Our thanks are also due to Dr. E. B. Hendry who allowed generous facilities for this work, Professor D. F. Cappell who permitted the use of equipment within his department, and Professor J. N. Davidson, F.R.S. and Dr. R. Y. Thomson for their advice and guidance.

### REFERENCES

ALLARD, C., DE LAMIRANDE, G. AND CANTERO, A.—(1953) *Can. J. med. Sci.*, **31**, 103.—(1957) *Cancer Res.*, **17**, 862.

ALLFREY, V. AND MIRSKY, A. E.—(1952) *J. gen. Physiol.*, **36**, 227.

BAUDHUIN, P.—(1959) *Archs int. Physiol. Biochim.*, **67**, 106.

BLUMENTHAL, G.—(1957) *J. Embryol. exp. Morph.*, **5**, 377.

BONHAM, D. G. AND GIBBS, D. F.—(1962) *Br. med. J.*, ii, 823.

BRODY, S. AND BALIS, M. E.—(1958) *Nature, Lond.*, **182**, 940.—(1959) *Cancer Res.*, **19**, 538.

BRODY, S. AND THORELL, B.—(1957) *Biochim. biophys. Acta*, **25**, 579.

CHAN, S. K., MCCOY, T. A. AND KIZER, D. E.—(1959) *Proc. Soc. exp. Biol. Med.*, **102**, 102.

COLEMAN, J. R.—(1963) *Biochim. biophys. Acta*, **68**, 141.

DAOUST, R. AND AMANO, H.—(1963) *Cancer Res.*, **23**, 131.

DICKMAN, S. R. AND MORRILL, G. A.—(1959) *Ann. N.Y. Acad. Sci.*, **81**, 585.

DOUNCE, A. L., O'CONNELL, M. P. AND MONTY, K. J.—(1957) *J. biophys. biochem. Cytol.*, **3**, 649.

DE DUVE, C., PRESSMAN, B. C., GIANETTO, R., WATTIAUX, R. AND APPELMANS, F.—(1955) *Biochem. J.*, **60**, 604.

DE DUVE, C., WATTIAUX, R. AND BAUDHUIN, P.—(1962) *Adv. Enzymol.*, **24**, 291.

EICHEL, H. J. AND ROTH, J. S.—(1962) *J. Cell. Biol.*, **12**, 263.

FESTY, B. AND PAOLETTI, C.—(1963) *C.r. hebd. Séanc. Acad. Sci., Paris*, **257**, 3682.

FIALA, S.—(1953) *Naturwissenschaften*, **40**, 391.—(1961) *Rass. Med. sper.*, **8**, 99.

FIALA, S. AND FIALA, A. E.—(1959) *Br. J. Cancer*, **13**, 136.

FIALA, S. AND KASINSKY, H. E.—(1961) *J. natn. Cancer Inst.*, **26**, 1059.

FODOR, P. J., TOMASHEFSKY, P. AND FUNK, C.—(1958) *Archs Biochem.*, **76**, 245.

GOLDBERG, D. M.—(1965) *Br. med. J.*, i, 353.

GOLDBERG, D. M., MACVICAR, J. AND WATTS, C.—(1967) *Clin. Sci.*, **32**, 71.

GOUTIER, R. AND GOUTIER-PIROTTE, M.—(1961) *Nature, Lond.*, **192**, 371.

GOUTIER, R. AND LEONARD, A.—(1962) *Expl Cell Res.*, **28**, 335.

GOUTIER-PIROTTE, M. AND GOUTIER, R.—(1962) *Radiat. Res.*, **16**, 728.

GOUTIER-PIROTTE, M. AND OTH, A.—(1956) *Biochim. biophys. Acta*, **22**, 394.

HENSTELL, H. H. AND FREEDMAN, R. I.—(1952) *Cancer Res.*, **12**, 341.

KEIR, H. M.—(1962) *Biochem. J.*, **85**, 265.

KEIR, H. M. AND GOLD, E.—(1963) *Biochim. biophys. Acta*, **72**, 263.

DE LAMIRANDE, G. AND ALLARD, C.—(1959*a*) *Ann. N.Y. Acad. Sci.*, **81**, 570.—(1959*b*) *Proc. Am. Ass. Cancer Res.*, **3**, 16.

DE LAMIRANDE, G., ALLARD, C. AND CANTERO, A.—(1954) *Can. J. Biochem. Physiol.*, **32**, 35.—(1958) *Cancer Res.*, **18**, 952.

de Lamirande, G., Allard, C., da Costa, H. C. and Cantero, A.—(1954) *Science, N.Y.*, **119**, 351.
Lawson, J. G.—(1959) *J. Obstet. Gynaec. Br. Commonw.* **66**, 946.—(1960) *J. Obstet. Gynaec. Br. Commonw.*, **67**, 305.
Ledoux, L., Brandli, S. and Paepe, J. C. de—(1958) *Nature, Lond.*, **181**, 913.
Ledoux, L., Pileri, A. and Vanderhaeghe, F.—(1958) *Archs int. Physiol. Biochim.*, **66**, 124.
Lindberg, U.—(1964) *Biochim. biophys. Acta*, **82**, 237.
Liss, M. and Lever, W. F.—(1962) *J. Invest. Derm.*, **39**, 529.
Loiselle, J-M. and Carrier, R.—(1963) *Can. J. Biochem. Physiol.* **41**, 2423.
Lowry, O. H., Rosebrough, N. J., Farr, A. L. and Randall, R. J.—(1951) *J. biol. Chem.*, **193**, 265.
Luibel, F. J., Sanders, E. and Ashworth, C. T.—(1960) *Cancer Res.*, **20**, 357.
Mantsavinos, R. and Cannelakis, E. S.—(1959) *J. biol. Chem.*, **234**, 628.
Martin, S. J., England, H., Turkington, V. and Leslie, I.—(1963) *Biochem. J.*, **89**, 327.
Maver, M. E. and Greco, A. E.—(1956) *J. natn. Cancer Inst.*, **17**, 503.
Morais, R. and de Lamirande, G.—(1965) *Biochim. biophys. Acta*, **95**, 40.
Morris, A. J. and Dickman, S. R.—(1960) *J. biol. Chem.*, **235**, 1404.
Odell, L. D. and Burt, J. C.—(1950) *J. Am. med. Ass.*, **142**, 226.
Reid, E. and Lewin, I.—(1957) *Br. J. Cancer*, **11**, 494.
Reid, E. and Lotz, F.—(1958) *Br. J. Cancer*, **12**, 419.
Reid, E. and Nodes, J. T.—(1959) *Ann. N.Y. Acad. Sci.*, **81**, 618.—(1963) *Nature, Lond.*, **199**, 176.
Reid, E. and Stevens, B. M.—(1958) *Biochem. J.*, **68**, 367.
Roth, J. S.—(1956) *Biochim. biophys. Acta*, **21**, 34.—(1960) *J. biophys. biochem. Cytol.*, **7**, 443.—(1962) *Biochim. biophys. Acta*, **61**, 903.—(1963) *Cancer Res.*, **23**, 657.
Roth, J. S. and Hilton, S.—(1963) *Radiat. Res.*, **19**, 42.
Roth, J. S., Hilton, S. and Morris, H. P.—(1964) *Cancer Res.*, **24**, 294.
Roth, J. S., Sheid, B. and Morris, H. P.—(1963) *Cancer Res.*, **23**, 454.
Russell, W. C., Gold, E., Keir, H. M., Omura, H., Watson, D. H. and Wildy, P.—(1964) *Virology*, **22**, 103.
Santoianni, P. and Rothman, S.—(1961) *J. invest. Derm.*, **37**, 489.
Sarkar, N. K.—(1961) *Archs Biochem.*, **93**, 328.
Schneider, W. C., Hogeboom, G. H., Shelton, E. and Striebich, M. J.—(1953) *Cancer Res.*, **13**, 285.
Shortman, K.—(1961) *Biochim. biophys. Acta*, **51**, 37.
Solomon, J. B.—(1960) *Biochem. J.*, **75**, 278.—(1964) *Nature, Lond.*, **201**, 618.
Steigleder, G. K. and Raab, W. P.—(1962) *J. invest. Derm.*, **38**, 209.
Waravdekar, V. S., Paradis, A. D. and Leiter, J.—(1955) *J. natn. Cancer Inst.*, **16**, 99.
Zytko, J., de Lamirande, G., Allard, C. and Cantero, A.—(1958) *Biochim. biophys. Acta*, **27**, 495.